# Pairwise correlation of genes involved in glucose metabolism: a potential diagnostic marker of cancer?

**DOI:** 10.18632/genesandcancer.216

**Published:** 2021-06-17

**Authors:** Meena Kishore Sakharkar, Karthic Rajamanickam, Shaoping Ji, Sarinder Kaur Dhillon, Jian Yang

**Affiliations:** ^1^Drug Discovery and Development Research Group, College of Pharmacy and Nutrition, University of Saskatchewan, Saskatoon, SK S7N 5E5, Canada; ^2^Henan Provincial Engineering Centre for Tumor Molecular Medicine, Institute of Molecular Medicine, School of Basic Medical Sciences, Henan University, Kaifeng, Henan Province 474004, P.R. of China; ^3^Institute of Biological Sciences, Faculty of Science, University of Malaya, Kuala Lumpur 50603, Malaysia

**Keywords:** gene expression, gene pair correlation, diagnostic marker, pan-cancer analysis, protein-protein interaction network

## Abstract

Cancer is a highly malignant disease, killing approximately 10 million people worldwide in 2020. Cancer patient survival substantially relies on early diagnosis. In this study, we evaluated whether genes involved in glucose metabolism could be used as potential diagnostic markers for cancer. In total, 127 genes were examined for their gene expression levels and pairwise gene correlations. Genes *ADH1B* and *PDHA2* were differentially expressed in most of the 12 types of cancer and five pairs of genes exhibited consistent correlation changes (from strong correlations in normal controls to weak correlations in cancer patients) across all types of cancer. Thus, the two differentially expressed genes and five gene pairs could be potential diagnostic markers for cancer. Further preclinical and clinical studies are warranted to prove whether these genes and/or gene pairs would indeed aid in early diagnosis of cancer.

## INTRODUCTION

Despite recent advances in diagnosis and treatment, cancer remains one of the top challenges to human health. Prognosis for advanced-stage and recurrent cancer remains poor. Cancer is highly heterogeneous and can remarkably reprogram metabolic pathways [1]. Metabolic reprogramming not only alters the type and concentration of intracellular and extracellular metabolites but also modulates gene expression and tumor microenvironment for cell proliferation and survival [2, 3]. However, pan-cancer analysis of metabolic reprogramming is limited except for observation of increased glucose uptake and aerobic glycolysis (Warburg Effect) in cancer cells [4, 5]. 

Glucose is the major energy source for cells, and thus, glucose metabolism is essential for normal cell functions and survival. In cancer cells, glucose metabolism is opted to low-efficient aerobic glycolysis over oxidative phosphorylation. This is likely an evolutionary adaptation to the hypoxic microenvironment. Furthermore, abnormality in glucose metabolism, such as the Krebs cycle, can trigger cancer metastasis and resistance towards chemotherapy [6-9]. Although there is a dispute on whether aerobic glycolysis is the cause or consequence of cancer, it is clear that cancer usually initiates in a hypoxic region and the switch to aerobic glycolysis is far ahead of cancer being diagnosed by lab tests or medical imaging [10-12]. Therefore, analysis of gene expression in the glucose metabolic pathway not only provides us valuable information on carcinogenesis but also could be used for early diagnosis of cancer. 

Early diagnosis remains crucial for cancer patient survival. Our previous study showed that gene expression correlation coefficient could be used as a prognostic/diagnostic biomarker for human breast cancer [13]. Recently, we also reported that loss of gene pair correlations in the sphingolipid metabolic pathway and tryptophan metabolic pathway could be a hallmark in cancer diagnosis [14, 15]. In the current study, we undertake a pan-cancer analysis of gene expression and gene pair correlation for 127 genes involved in glucose metabolism using The Cancer Genome Atlas (TCGA).

**Figure 1:**
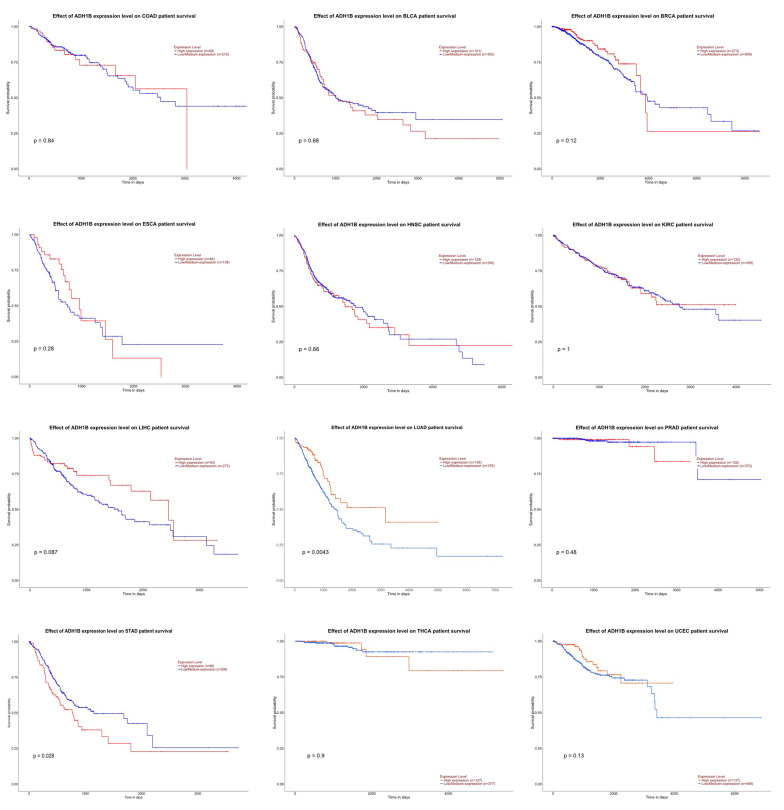
Kaplan-Meier plots of *ADH1B* in 12 different types of cancer. Patients with high expression of *ADH1B* were shown in red and patients with low/medium expression of *ADH1B* were shown in blue, respectively. *ADH1B* is a favorable prognostic factor for lung adenocarcinoma (LUAD, *p* = 0.004) but an unfavorable prognostic factor for stomach adenocarcinoma (STAD, *p* = 0.028). The Kaplan-Meier plots were generated using online software UALCAN (http://ualcan.path.uab.edu, accessed on May 23, 2021).

**Table 1:** Table 1: Ten genes showing similar trend of expression change (either upregulated or downregulated) across all 12 types of cancer, with the two genes differentially expressed (|log_2_FC| ≥ 1.00) in most of the 12 types of cancer highlighted in red.

Gene	BLCA	BRCA	COAD	ESCA	HNSC	KIRC	LIHC	LUAD	PRAD	STAD	THCA	UCEC
*ADH1B*	-4.40	-4.86	-4.06	-2.15	-3.41	-2.67	-1.16	-3.19	-1.66	-1.83	-3.44	-7.03
*ALDH2*	-1.64	-2.10	-0.26	-0.17	-0.65	-0.75	-1.02	-1.19	-0.81	-0.23	-0.84	-0.36
*ENO1*	0.96	0.33	1.00	1.48	1.14	0.64	1.27	1.22	0.19	1.12	0.17	1.53
*EPM2A*	-2.45	-0.96	-1.21	-0.79	-0.99	-1.30	-0.30	-1.17	-0.94	-1.10	-0.82	-2.00
*GAPDH*	0.67	1.05	0.90	1.29	0.56	1.50	1.35	1.72	0.08	0.64	0.44	1.45
*LDHA*	0.62	0.56	0.72	1.50	1.31	1.71	0.05	1.36	0.42	0.85	0.07	1.38
*MPC1*	-0.62	-0.38	-0.96	-0.69	-0.82	-1.56	-0.85	-0.37	-0.19	-0.42	-0.79	-0.38
*PDHA2*	N/A	1.13	1.88	2.99	1.65	2.27	3.72	3.43	0.73	2.92	1.35	1.73
*PFKFB4*	2.39	1.26	0.84	1.15	1.65	2.45	2.43	0.81	0.21	0.11	0.18	2.33
*PHKG2*	0.71	1.12	0.48	0.95	1.01	0.28	0.86	0.86	0.26	0.96	0.31	1.01

## RESULTS AND DISCUSSION 

Cancer is a heterogenous and complex disease, killing approximately 10 million people globally in 2020 [16]. Although recent advances, such as CAR T-cell therapy and immune checkpoint inhibitors, have provided more options for cancer treatment, prognosis for advanced-stage and recurrent cancer remains poor. For example, the 5-year survival rate for colon cancer drops from 92% for stage I down to 12% for stage IV in Canada [17]. Thus, early diagnosis is critical for patient’s survival. Gene expression profiling using microarray and RNA-Seq data has been widely used to identify diagnostic or prognostic gene signatures, such as the 70-Gene Signature Assay, which are differentially expressed between cancer patients and normal controls. Moreover, these specific gene signatures may help in identifying drug design targets for cancer treatment. 

Glucose metabolism is essential for normal cellular functions and cell growth, and switch to aerobic glycolysis (Warburg Effect) has been recognized as a characteristic of cancer. Thus, we undertook a pan-cancer analysis of 127 genes involved in glucose metabolism in 12 cancer datasets which meet the selection criteria of N_normal_ ≥ 10 and N_cancer_ ≥ 10. The 12 types of cancer are bladder urothelial carcinoma (BLCA), breast invasive carcinoma (BRCA), colon adenocarcinoma (COAD), esophageal carcinoma (ESCA), head and neck squamous cell carcinoma (HNSC), kidney renal clear cell carcinoma (KIRC), liver hepatocellular carcinoma (LIHC), lung adenocarcinoma (LUAD), prostate adenocarcinoma (PRAD), stomach adenocarcinoma (STAD), thyroid carcinoma (THCA), and uterine corpus endometrial carcinoma (UCEC). For each gene, the normalized log2FC in gene expression between cancer patients and normal controls was calculated and presented in Supplementary Table S1. As shown in Table 1, there were 10 genes showing similar trend of gene expression change (either upregulated or downregulated) across all 12 types of cancer. These genes are *ADH1B*, *ALDH2*, *ENO1*, *EPM2A*, *GAPDH*, *LDHA*, *MPC1*, *PDHA2*, *PFKFB4* and *PHKG2*. However, only one gene, *ADH1B* (highlighted in red in Table 1) was differentially downregulated in all types of cancer upon using |log_2_FC| ≥ 1.00 and p < 0.05 as the cut-off. This implicated that *ADH1B* could be applied as a diagnostic marker for cancer. *ADH1B* encodes alcohol dehydrogenase 1B, which catalyzes the oxidation of alcohol to form acetaldehyde. *ADH1B* is downregulated in hepatocellular carcinoma [18] and its polymorphism is associated with increased risk for various types of cancer, such as colorectal cancer, gastric cancer, and esophageal cancer [19-21]. We further performed survival analysis and generated Kaplan-Meier plots for *ADH1B* for the 12 types of cancer (Figure 1). *ADH1B* is a favorable prognostic factor for lung adenocarcinoma (LUAD, *p* = 0.004) but an unfavorable prognostic factor for stomach adenocarcinoma (STAD, *p* = 0.028). Other than *ADH1B*, gene *PDHA2* (also highlighted in red in Table 1) was differentially upregulated in most types of cancer except in bladder urothelial carcinoma (BLCA, data not available) and prostate adenocarcinoma (PRAD, log_2_FC = 0.73), implicating that *PDHA2* could also be used as a diagnostic marker. *PDHA2* encodes subunit alpha 2 of pyruvate dehydrogenase 1E, which is a major component of the pyruvate dehydrogenase complex (PDC) catalyzing the oxidative decarboxylation of pyruvate to form acetyl-CoA. *PDHA2* is predominately expressed in germ cells, whereas its homologue, *PDHA1*, is expressed in somatic cells [22]. It has been shown that downregulation of *PDHA1* promotes cancer progression and acts as a poor prognostic factor for cancer [23-26]. However, the biological function of *PDHA2* has barely been studied in cancer cells. Only recently, Lv et al. proposed gene pair *PDHA2-APRT* as a potential prognostic marker for breast cancer patients after treatment with tamoxifen [27].

**Figure 2:**
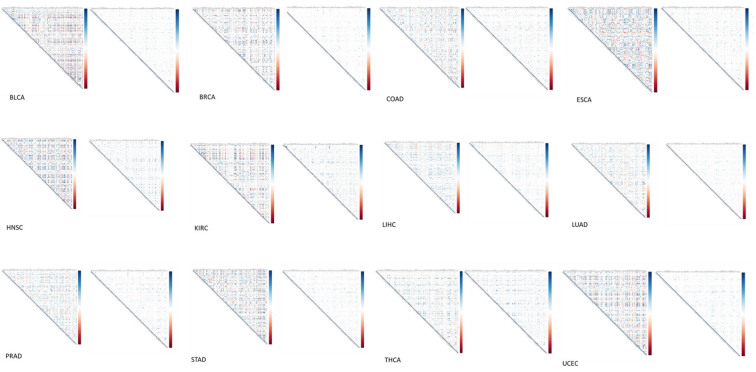
Gene pair correlations of 127 genes involved in glucose metabolism across 12 different types of cancer (BLCA, BRCA, COAD, ESCA, HNSC, KIRC, LIHC, LUAD, PRAD, STAD, THCA and UCEC). Positive and negative correlations are represented by blue and red dots, respectively, and the sizes of the dots are proportional to the correlation coefficient values.

**Figure 3:**
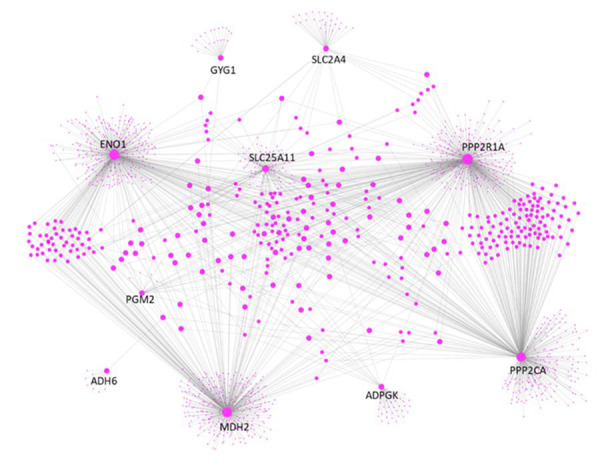
The protein-protein interaction (PPI) network for proteins encoded by the 5 pairs of genes with correlation changes consistent across all 12 types of cancer.

**Table 2:** Table 2: Five pairs of genes with correlation changes consistent across all 12 types of cancer.

Gene pair	Change of pairwise correlation from normal to cancer
*ADH6 – GYC1*	Negative → Less negative
*ADPGK – SLC2A4*	Negative → Less negative
*ENO1 – PPP2R1A*	Positive → Less positive
*MDH2 – SLC25A11*	Positive → Less positive
*PGM2 – PPP2CA*	Positive → Less positive

Some gene signatures, especially single gene makers, have failed to serve as diagnostic or prognostic markers, because alterations of their expressions are not sufficient enough to be detected [28]. Carcinogenesis is a very complex process, which requires the coordination of multiple genes. Increased/decreased correlations among genes are highly likely happening prior to expression changes of the individual genes. Our previous studies have shown that pairwise correlation coefficients were dramatically decreased for genes involved in either sphingolipid metabolism or tryptophan metabolism in cancer patients as compared to normal controls [14, 15]. Due to the crucial role glucose metabolism in normal cellular functions, we decided to calculate the pairwise gene correlation coefficients for the 127 genes involved in glucose metabolism. Glucose metabolism genes were widely and strongly correlated in normal controls, however, the gene-pair correlation coefficients were significantly decreased or lost in cancer patients for all 12 types of cancer (Figure 2). In addition, we examined whether any pair of genes exhibited consistent correlation change across all types of cancer in one of the following six correlation categories: positive → more positive, positive → less positive, positive → negative, negative → more negative, negative → less negative and negative → positive. Only 5 pairs of genes were identified (Table 2). Interestingly, the correlation became weaker for all 5 pairs of genes upon carcinogenesis, with *ADH6* – *GYC1* and *ADPGK* – *SLC2A4* changed from negatively correlated in normal controls to less negatively correlated in cancer patients and *ENO1* – *PPP2R1A*, *MDH2* – *SLC25A11* and *PGM2* – *PPP2CA* changed from positively correlated in normal controls to less positively correlated in cancer patients. Thus, correlation coefficients for these 5 pairs of genes could be applied as a potential diagnostic maker for cancer and/or an indicator of cancer prevalence in a community when compared with a normal control. However, the differentially downregulated gene *ADH1B* and differentially upregulated gene *PDHA2* were not present in the identified gene pairs. This implicates that genes may decouple even without significantly altering their respective expression level upon carcinogenesis. The decoupling of genes might help to diagnose cancer at a much earlier stage than the currently used diagnostic techniques, such as gene signature and cancer antigen assays, which depend on changes of gene or protein levels. 

Finally, we constructed the PPI network for proteins encoded by the 5 pairs of genes. As illustrated in Figure 3, the 10 proteins, especially ENO1, MDH2, PPP2R1A and PPP2CA, are hubs in the PPI network. They make 1513 direct interactions with other proteins, including 48 proteins involved in glucose metabolism. Because biological hubs are normally drug development targets, further studies are warranted to identify whether these 10 proteins could also be the intervention sites for cancer treatment.

## Materials and Methods

### Data acquisition

A list of 127 genes involved in glucose metabolism was downloaded from PathCards (https://pathcards.genecards.org/), which is an integrated database of human biological pathways and their annotations. Cancer RNA-Seq datasets (N_normal _≥ 10 and N_cancer_ ≥ 10) were downloaded from TCGA via the Genomic Data Commons (GDC) data portal. In total, 543 normal controls and 5641 cancer patients from 12 different types of cancer were involved in the study. The numbers of cancer patients and normal controls for each type of cancer were summarized in Table 3. For each dataset, 60,483 RNA transcripts were analyzed in term of FPKM value. 

**Table 3:** Table 3: Numbers of normal controls and cancer patients in 12 different types of cancer.

Type	Cancer description	Normal	Cancer
BLCA	Bladder urothelial carcinoma	19	414
BRCA	Breast invasive carcinoma	113	1102
COAD	Colon adenocarcinoma	41	471
ESCA	Esophageal carcinoma	11	159
HNSC	Head and neck squamous cell carcinoma	13	127
KIRC	Kidney renal clear cell carcinoma	72	538
LIHC	Liver hepatocellular carcinoma	50	371
LUAD	Lung adenocarcinoma	59	533
PRAD	Prostate adenocarcinoma	52	498
STAD	Stomach adenocarcinoma	32	375
THCA	Thyroid carcinoma	58	502
UCEC	Uterine corpus endometrial carcinoma	23	551

### Identification and visualization of differentially expressed genes

The protocol on identifying differentially expressed genes (DEGs) in cancer against normal using DEGseq in the R package has been published [14, 15]. Briefly, Likelihood Ratio Test (LRT) was applied, and sample expression profiles were screened using *p*-value < 0.05. The output was expressed in normalized log_2_fold-change (Log_2_FC). Then, expression changes of the 127 genes involved in glucose metabolism were extracted for the 12 types of cancer (Supplementary Table S1). 

### Computation and visualization of correlation matrix

For each type of cancer, correlation matrix was calculated using cor function and visualized using corrplot function in the R package. Positive and negative correlations are represented in blue and red, respectively.

### Protein-protein interaction (PPI) network

Human protein interactome (BIOGRID-ORGANISM-Homo_sapiens-4.0.189.tab) was downloaded from the BioGRID database [29]. PPI data were then extracted from the protein interactome and plotted using Cytoscape [30] for genes involved in the 5 gene pairs which were conserved across all 12 types of cancer.

## CONCLUSION

In this study, we evaluated the expression and gene pair correlation for 127 genes involved in glucose metabolism across 12 different types of cancer. Genes *ADH1B* and *PDHA2* were differentially expressed in most of the 12 types of cancer. We also identified five pairs of genes having consistent correlation changes (weaker correlations in cancer patients) in all types of cancer. The two differentially expressed genes and five gene pairs could be potential diagnostic markers for cancer. 

## SUPPLEMENTARY MATERIALS


