# Supplementary material for: Pairwise correlation of genes involved in glucose metabolism: a potential diagnostic marker of cancer?
Source: Genes Cancer. 2021 Jun 17;12:69–76. doi: 10.18632/genesandcancer.216 (PMC8211569; doi:10.18632/genesandcancer.216)
Supplement: Supplementary file 1 [file ganc-12-216-s001.pdf]

Table S1: Expression change (log_2FC) between normal controls and cancer patients for 127 genes involved in glucose metabolism across 12 different types of cancer. The 12 types of cancer are bladder urothelial carcinoma (BLCA), breast invasive carcinoma (BRCA), colon adenocarcinoma (COAD), esophageal carcinoma (ESCA), head and neck squamous cell carcinoma (HNSC), kidney renal clear cell carcinoma (KIRC), liver hepatocellular carcinoma (LIHC), lung adenocarcinoma (LUAD), prostate adenocarcinoma (PRAD), stomach adenocarcinoma (STAD), thyroid carcinoma (THCA), and uterine corpus endometrial carcinoma (UCEC).

GENE	BLCA	BRCA	COAD	ESCA	HNSC	KIRC	LIHC	LUAD	PRAD	STAD	THCA	UCEC
ACSS1	-0.04	0.52	0.70	-0.50	0.25	-0.74	1.71	-0.27	0.78	0.69	-0.68	-0.23
ACSS2	0.36	-1.57	-0.82	0.02	0.00	-0.65	0.51	-0.61	-0.31	0.03	-0.18	-0.54
ADH1A	-0.39	-4.50	-1.91	-3.33	-1.33	2.18	-1.14	-1.77	0.47	1.57	-2.01	-2.25
ADH1B	-4.40	-4.86	-4.06	-2.15	-3.41	-2.67	-1.16	-3.19	-1.66	-1.83	-3.44	-7.03
ADH1C	-1.65	-3.38	-3.91	-1.08	-1.77	-3.41	-0.92	2.05	-0.42	-1.21	-2.87	0.13
ADH4	1.54	-3.67	0.51	-1.72	-3.10	1.88	-1.71	2.72	-0.80	-1.73	-1.02	0.64
ADH5	-0.49	-0.86	-0.38	0.37	1.18	-0.52	0.26	-0.28	-0.54	0.19	-0.70	-0.59
ADH6	1.27	-1.20	-1.10	-1.26	-1.35	-2.50	-0.85	0.79	-0.01	-0.24	-1.21	-1.06
ADH7	-1.08	-0.70	-0.38	0.43	-1.49	3.99	0.61	0.76	-1.43	-3.82	1.68	3.68
ADPGK	0.83	0.31	0.62	0.88	1.37	0.93	0.96	-0.11	0.52	1.40	-0.03	0.47
AGL	-0.53	0.23	-0.44	0.26	-0.98	-0.92	-0.76	0.39	-0.49	0.45	-0.65	-0.29
AKR1A1	0.53	0.53	0.30	0.35	0.17	-0.28	0.29	0.41	0.50	0.13	0.07	0.72
ALDH1A3	-1.07	-1.74	0.72	1.05	-0.41	-1.44	-0.10	0.27	0.94	0.89	2.56	-1.47
ALDH1B1	-2.41	0.74	0.78	0.05	0.88	-1.17	-0.64	1.10	-0.56	0.82	-1.04	-1.96
ALDH2	-1.64	-2.10	-0.26	-0.17	-0.65	-0.75	-1.02	-1.19	-0.81	-0.23	-0.84	-0.36
ALDH3A1	0.87	-0.79	0.16	-0.35	-0.89	1.01	5.98	1.83	-1.53	-2.25	0.15	1.03
ALDH3A2	-0.24	-0.56	-0.40	0.45	-0.45	-0.60	0.41	-0.04	-0.50	-0.43	0.21	-0.06
ALDH3B1	-0.16	0.47	0.53	-0.15	-1.14	0.81	1.38	-0.86	-0.65	0.77	2.40	-1.40
ALDH3B2	0.59	0.42	3.73	1.28	0.53	-3.83	5.10	2.85	0.95	-1.28	4.14	2.57
ALDH7A1	-0.42	-0.57	0.17	0.43	-0.04	-0.64	0.22	0.23	0.05	0.39	-0.78	0.08
ALDH9A1	-0.48	-0.26	-0.17	0.17	-0.68	-0.67	-0.17	-0.18	-0.25	0.06	-0.58	-0.35
ALDOA	0.44	1.08	0.25	1.00	-0.04	1.13	1.92	1.20	-0.16	0.33	-0.01	0.82
ALDOB	2.87	-0.11	-3.14	-2.46	2.16	-4.64	-0.89	1.96	0.94	-3.79	0.63	-1.35
ALDOC	0.41	-1.85	1.02	0.52	0.73	2.31	0.41	0.80	-0.66	-0.80	-0.49	-0.37
BPGM	1.45	0.35	0.06	1.29	1.81	-0.40	1.40	0.23	-0.10	0.41	0.34	0.71

CALM1	-0.58	-0.29	-0.61	0.22	0.63	-0.28	0.16	-0.72	-0.79	-0.28	-0.06	-0.21
CALM2	-0.29	0.39	-0.36	0.98	0.80	-0.26	0.85	0.05	-0.10	0.33	0.18	-0.69
CALM3	-0.26	0.39	-0.34	0.69	0.97	0.51	1.01	-0.08	-0.11	0.21	-0.10	-0.36
DLAT	0.02	-0.08	-0.02	0.50	-0.14	-0.67	1.04	0.44	-0.14	0.79	-0.27	-0.01
DLD	-0.20	-0.23	-0.19	0.40	0.06	-0.88	0.43	0.12	-0.12	0.59	-0.40	-0.12
ENO1	0.96	0.33	1.00	1.48	1.14	0.64	1.27	1.22	0.19	1.12	0.17	1.53
ENO2	0.47	1.48	0.66	1.38	2.87	3.23	1.74	0.85	-0.93	0.29	-0.23	0.48
ENO3	0.19	-3.18	1.07	1.04	-3.17	0.55	-0.98	2.52	-0.13	0.89	0.64	0.72
ENO4	-0.37	0.25	0.03	0.13	-2.56	-0.43	0.84	-1.16	0.05	0.22	-1.45	-0.46
EPM2A	-2.45	-0.96	-1.21	-0.79	-0.99	-1.30	-0.30	-1.17	-0.94	-1.10	-0.82	-2.00
FBP1	-0.70	1.09	-0.22	-0.42	0.74	-2.21	-1.40	-1.61	0.70	-1.11	0.08	1.52
FBP2	0.29	-3.89	0.03	-2.99	-3.71	-1.42	1.30	-0.33	-3.19	-2.33	-2.33	3.78
G6PC	2.78	0.05	-6.02	-1.07	1.45	-3.35	-0.34	6.06	2.15	-4.32	-0.89	-0.85
G6PC2	-1.00	-0.61	-0.61	-2.34	-1.85	1.43	0.75	-2.38	0.01	-0.30	0.44	-1.97
G6PC3	0.65	0.74	0.91	0.90	0.63	-0.15	1.49	0.27	0.13	0.94	0.13	0.61
GAA	0.61	0.67	-0.10	1.12	1.13	0.37	0.88	-0.24	0.49	0.57	-0.08	0.46
GALM	0.53	0.26	-0.67	0.31	0.43	-1.09	0.24	0.46	-0.66	0.57	-0.27	-0.32
GAPDH	0.67	1.05	0.90	1.29	0.56	1.50	1.35	1.72	0.08	0.64	0.44	1.45
GAPDHS	2.87	1.59	1.79	2.32	-2.01	1.55	1.41	1.71	2.96	1.34	1.27	0.12
GBE1	-0.26	-1.37	0.22	0.37	-0.16	-0.16	0.10	0.05	-0.11	0.57	-0.49	-0.22
GCK	-0.68	-0.94	-0.76	1.55	1.99	0.03	2.62	0.41	-1.11	0.31	-0.84	-0.48
GNPDA1	0.16	0.48	1.15	1.33	1.22	-0.06	1.52	0.40	-0.15	1.18	0.44	0.20
GNPDA2	-0.40	-0.29	0.27	0.13	0.62	-0.09	1.16	-0.07	-0.21	0.23	-0.43	-1.14
GOT1	0.01	0.47	-0.54	-0.05	-0.95	-1.00	-0.35	1.00	-0.22	0.19	-0.17	0.83
GOT2	0.02	0.67	0.48	0.73	-0.08	-1.05	-0.32	0.56	-0.17	0.58	-0.19	0.06
GPI	0.53	0.57	0.82	1.21	0.60	0.57	1.13	1.13	0.35	0.92	-0.01	1.23
GYG1	-0.91	0.13	-0.03	0.66	0.04	0.11	1.07	-0.02	-0.36	-0.14	0.12	-1.07
GYG2	-0.37	-3.90	1.14	-0.01	0.39	-1.16	0.15	2.22	-0.02	1.35	-0.42	0.16
GYS1	0.33	-0.13	0.07	0.69	0.05	0.92	1.25	0.28	0.10	0.40	-0.04	0.45
GYS2	-3.02	-4.62	0.03	-2.52	-2.42	0.72	-1.53	1.21	-0.28	-4.07	-1.65	-0.12
HK1	-0.50	0.58	-0.02	0.22	-0.04	-0.01	1.12	0.00	-0.36	-0.34	-0.90	0.17
HK2	0.58	0.10	-1.04	1.23	1.09	3.48	2.35	-0.24	0.61	1.52	0.77	1.41

HK3	1.04	0.59	0.67	1.41	3.58	2.66	-0.61	-1.72	0.56	1.86	0.84	1.57
HKDC1	1.05	0.75	0.83	1.53	3.56	0.58	2.43	1.71	-0.29	0.93	-1.42	1.34
LDHA	0.62	0.56	0.72	1.50	1.31	1.71	0.05	1.36	0.42	0.85	0.07	1.38
LDHAL6A	2.04	-0.53	0.30	0.16	0.61	-0.37	1.48	0.38	-0.80	1.42	-1.39	-0.76
LDHAL6B	-0.65	-0.34	-0.05	0.85	1.89	0.82	0.29	0.83	-0.52	1.75	-0.80	0.44
LDHB	-0.06	-0.90	1.20	0.24	0.49	-1.50	0.98	0.90	-0.72	0.40	-0.37	0.23
LDHC	1.71	0.99	0.05	1.86	1.57	-1.39	0.94	0.88	0.44	1.42	-0.65	0.19
MDH1	-0.06	-0.45	-0.25	0.03	-0.11	-0.78	0.77	0.15	-0.20	0.11	0.04	0.05
MDH2	0.25	0.54	0.51	0.94	-0.12	-0.34	0.86	0.72	0.43	0.67	-0.08	0.34
MINPP1	0.62	0.37	-0.06	0.84	1.58	0.15	0.30	0.44	0.10	1.01	-0.17	0.15
MPC1	-0.62	-0.38	-0.96	-0.69	-0.82	-1.56	-0.85	-0.37	-0.19	-0.42	-0.79	-0.38
MPC2	-0.02	0.76	-0.27	-0.29	-0.05	-0.06	1.14	0.40	0.77	0.36	0.30	0.50
NHLRC1	0.90	0.78	0.44	1.57	1.06	0.17	1.39	0.87	0.53	1.21	-0.16	0.22
PC	0.64	-1.37	-0.89	1.04	1.07	-1.32	-0.28	1.73	-0.43	0.01	1.42	0.37
PCK1	1.90	-4.99	-2.66	1.31	-1.34	-2.69	-1.56	3.35	-0.42	-2.17	-1.15	0.72
PCK2	1.13	0.60	-0.70	1.06	0.96	-2.16	-0.59	-0.20	-0.17	-1.55	0.15	1.49
PDHA1	0.13	-0.19	0.25	0.00	-0.16	-1.13	0.62	0.30	-0.09	0.68	-0.18	0.42
PDHA2	N/A	1.13	1.88	2.99	1.65	2.27	3.72	3.43	0.73	2.92	1.35	1.73
PDHB	-0.10	0.18	-0.04	-0.04	-0.23	-1.12	0.52	0.05	0.00	0.26	-0.09	0.12
PDHX	-0.04	0.28	0.17	0.22	0.16	-0.42	0.43	0.35	0.04	0.97	-0.23	0.52
PFKFB1	0.92	-3.34	0.12	0.91	-1.87	-1.03	-0.03	0.42	0.24	1.07	-1.60	1.69
PFKFB2	0.55	0.51	-0.34	0.19	0.54	-1.66	2.00	-0.56	0.42	1.46	-1.27	1.34
PFKFB3	-0.41	-1.80	0.88	0.79	1.09	-1.24	0.18	-0.44	-0.97	0.64	0.11	-0.40
PFKFB4	2.39	1.26	0.84	1.15	1.65	2.45	2.43	0.81	0.21	0.11	0.18	2.33
PFKL	0.42	0.56	-0.04	0.22	0.63	0.34	0.72	0.34	0.25	0.28	0.11	0.81
PFKM	-0.72	-0.31	0.77	0.46	-1.53	-0.94	1.49	0.03	-0.25	0.12	-0.34	-1.40
PFKP	0.04	0.71	0.44	1.50	0.73	2.02	2.43	2.17	-0.57	0.93	0.11	0.27
PGAM1	0.09	0.39	0.08	0.86	0.96	0.70	0.44	0.48	-0.48	-0.01	-0.18	0.26
PGAM2	N/A	N/A	N/A	N/A	N/A	N/A	N/A	N/A	N/A	N/A	N/A	N/A
PGAM4	-0.13	0.32	0.57	0.27	0.75	0.45	0.59	0.55	-0.41	-0.11	-0.61	0.33
PGK1	0.70	0.86	0.94	1.34	1.16	0.96	1.13	0.80	-0.22	0.90	-0.55	0.76
PGK2	1.02	4.65	2.63	1.88	N/A	1.19	5.97	2.45	1.85	1.65	-0.11	2.43

PGM1	-0.28	-1.08	-1.26	-0.01	-0.56	0.35	-0.54	-0.61	-0.49	-0.03	-0.03	0.13
PGM2	0.30	0.32	0.52	0.82	0.21	0.08	0.45	0.19	0.04	0.48	-0.55	-0.51
PHKA1	-0.16	0.45	1.60	-0.13	-0.52	0.72	0.50	1.66	0.15	0.75	0.29	-0.09
PHKA2	0.75	-0.46	0.35	0.40	0.35	2.27	0.66	0.12	0.41	0.99	-0.25	-0.41
PHKB	-0.62	-0.33	-0.17	-0.11	-0.25	-0.65	0.66	0.09	0.12	0.26	-0.64	-0.33
PHKG1	-1.35	-0.70	-0.59	0.62	-1.72	2.04	1.56	0.04	0.04	0.89	-0.36	-0.41
PHKG2	0.71	1.12	0.48	0.95	1.01	0.28	0.86	0.86	0.26	0.96	0.31	1.01
PKLR	3.17	0.96	0.43	0.96	1.75	-0.72	0.59	2.38	0.38	-0.62	-0.25	0.93
PKM	0.58	0.93	1.15	1.34	1.26	0.60	2.76	0.92	-0.26	1.23	0.46	1.27
PPP1R3C	-0.72	-0.07	-1.88	-1.28	-2.35	2.01	0.16	-0.59	-1.65	-2.13	0.19	-3.75
PPP2CA	-0.20	0.28	0.20	0.78	0.46	-0.02	0.72	-0.02	0.01	0.55	-0.25	-0.20
PPP2CB	-0.72	-0.43	-0.55	0.48	0.11	-0.42	-0.06	-0.79	-0.41	0.16	-0.50	-1.20
PPP2R1A	0.17	0.57	0.22	0.60	0.40	-0.13	1.23	0.17	0.10	0.42	0.22	0.13
PPP2R1B	0.49	-1.35	0.24	-0.05	0.68	-0.32	0.57	0.56	0.34	0.95	-0.59	0.61
PPP2R5D	0.26	0.30	0.12	1.11	0.89	0.02	1.29	0.30	0.00	0.67	-0.25	-0.12
PRKACA	-0.04	-0.01	-0.36	0.60	0.07	0.10	0.47	-0.38	-0.29	0.15	0.33	-0.46
PRKACB	-1.01	0.76	-1.76	-1.33	0.16	-0.47	0.44	-0.02	-0.04	-0.16	-0.63	-1.43
PRKACG	-0.43	0.07	0.74	0.15	N/A	0.40	0.10	-1.30	-1.58	2.50	-0.46	-0.09
PYGB	-1.58	-0.39	0.35	0.57	0.90	-0.40	2.22	0.70	-0.39	0.87	0.24	-0.14
PYGL	0.38	-1.10	0.01	1.51	2.35	2.08	0.21	0.64	-1.06	0.20	0.04	0.53
PYGM	-4.49	-5.75	-3.48	-1.77	-3.95	0.72	1.11	-1.15	-1.34	-2.44	-0.78	-3.24
RPS27A	-0.21	-0.46	0.63	0.56	0.27	0.57	1.20	0.21	0.42	0.29	-0.43	-0.64
SLC25A1	0.95	-0.01	0.00	0.91	1.03	0.31	0.75	0.40	0.39	0.23	-0.25	0.92
SLC25A10	1.08	1.17	0.68	0.84	0.11	-0.98	0.65	2.20	1.08	1.07	0.07	2.42
SLC25A11	0.20	0.18	-0.29	0.72	-0.02	-0.42	0.51	-0.13	-0.04	0.18	0.14	0.15
SLC25A12	-0.86	-0.27	-0.30	0.19	-0.42	-0.57	1.50	0.36	-0.72	-0.26	-0.46	-0.70
SLC25A13	0.74	0.36	0.15	1.15	0.87	-0.30	0.14	1.11	0.19	1.33	-0.03	1.00
SLC2A1	0.41	1.45	1.96	1.67	2.34	2.02	2.01	3.52	-0.46	1.27	0.40	1.79
SLC2A2	4.52	1.21	-4.56	6.17	4.12	0.52	-0.46	6.99	2.31	-3.06	-0.03	2.16
SLC2A3	-1.54	-0.80	1.16	1.30	1.55	2.09	-0.18	-1.04	-0.22	0.79	0.04	-1.64
SLC2A4	-4.59	-4.31	-2.41	-2.06	-3.09	0.34	1.10	-0.36	-1.21	-2.07	-0.26	-3.86
SLC2A5	0.31	-0.19	-1.26	1.44	0.65	1.83	5.07	2.49	-1.87	-2.53	-0.57	0.25

SLC37A4	1.27	0.36	0.06	0.47	0.50	0.89	-0.04	0.89	-0.51	-0.06	0.18	0.60
TPI1	0.72	0.97	0.47	1.20	0.92	1.05	0.84	1.13	0.11	0.57	-0.10	1.48
UBA52	0.02	0.14	0.78	0.80	0.21	0.63	1.08	0.06	0.40	0.19	-0.04	0.25
UBB	-0.13	0.30	-0.35	0.55	0.18	-0.41	0.24	-0.36	0.06	0.22	-0.33	0.18
UBC	-0.72	-0.08	0.07	0.65	0.20	0.88	0.55	-0.17	-0.25	0.04	-0.51	-0.18
UGP2	-0.59	-1.16	-1.88	0.24	-0.40	-0.21	-0.44	0.23	-0.37	0.23	-0.30	-0.91
